# m^6^A methylation reader IGF2BP2 activates endothelial cells to promote angiogenesis and metastasis of lung adenocarcinoma

**DOI:** 10.1186/s12943-023-01791-1

**Published:** 2023-06-23

**Authors:** Han Fang, Qi Sun, Jin Zhou, Huijuan Zhang, Qiong Song, Hua Zhang, Guohua Yu, Ying Guo, Chengyu Huang, Yakui Mou, Chuanliang Jia, Yingjian Song, Aina Liu, Kaiyu Song, Congxian Lu, Ruxian Tian, Shizhuang Wei, Dengfeng Yang, Yixuan Chen, Ting Li, Kejian Wang, Yilan Yu, Yufeng Lv, Ke Mo, Ping Sun, Xiaofeng Yu, Xicheng Song

**Affiliations:** 1 Department of Otorhinolaryngology, Head and Neck Surgery, Yantai Yuhuangding Hospital of Qingdao University, Yantai, 264000 Shandong China; 2 Key Laboratory of Spatiotemporal Single-Cell Technologies and Translational Medicine, Yantai, 264000 Shandong China; 3 Department of Endocrinology, Yantai Yuhuangding Hospital of Qingdao University, Yantai, 264000 Shandong China; 4 Department of Oncology, Yantai Yuhuangding Hospital of Qingdao University, Yantai, 264000 Shandong China; 5 Department of Pathology, Yantai Yuhuangding Hospital of Qingdao University, Yantai, 264000 Shandong China; 6 Shandong Provincial Clinical Research Center for Otorhinolaryngologic Diseases, Yantai, 264000 Shandong China; 7 Department of Thoracic Surgery, Yantai Yuhuangding Hospital of Qingdao University, Yantai, 264000 Shandong China; 8 Biology Institute, Guangxi Academy of Sciences, Nanning, 530007 Guangxi China; 9 Department Of Basic Science, YuanDong Life California Ivy Research Institute, West Hollywood, CA 90069 USA; 10 Experimental Center of BIOQGene, YuanDong International Academy Of Life Sciences, Hong Kong, 999077 China

**Keywords:** Lung adenocarcinoma, Single-cell RNA sequencing, Metastasis, N^6^-methyladenosine, Angiogenesis, IGF2BP2, Exosomes

## Abstract

**Background:**

Lung adenocarcinoma (LUAD) is a common type of lung cancer with a high risk of metastasis, but the exact molecular mechanisms of metastasis are not yet understood.

**Methods:**

This study acquired single-cell transcriptomics profiling of 11 distal normal lung tissues, 11 primary LUAD tissues, and 4 metastatic LUAD tissues from the GSE131907 dataset. The lung multicellular ecosystems were characterized at a single-cell resolution, and the potential mechanisms underlying angiogenesis and metastasis of LUAD were explored.

**Results:**

We constructed a global single-cell landscape of 93,610 cells from primary and metastatic LUAD and found that IGF2BP2 was specifically expressed both in a LUAD cell subpopulation (termed as LUAD_IGF2BP2), and an endothelial cell subpopulation (termed as En_IGF2BP2). The LUAD_IGF2BP2 subpopulation progressively formed and dominated the ecology of metastatic LUAD during metastatic evolution. IGF2BP2 was preferentially secreted by exosomes in the LUAD_IGF2BP2 subpopulation, which was absorbed by the En_IGF2BP2 subpopulation in the tumor microenvironment. Subsequently, IGF2BP2 improved the RNA stability of FLT4 through m^6^A modification, thereby activating the PI3K-Akt signaling pathway, and eventually promoting angiogenesis and metastasis. Analysis of clinical data showed that IGF2BP2 was linked with poor overall survival and relapse-free survival for LUAD patients.

**Conclusions:**

Overall, these findings provide a novel insight into the multicellular ecosystems of primary and metastatic LUAD, and demonstrate that a specific LUAD_IGF2BP2 subpopulation is a key orchestrator promoting angiogenesis and metastasis, with implications for the gene regulatory mechanisms of LUAD metastatic evolution, representing themselves as potential antiangiogenic targets.

**Supplementary Information:**

The online version contains supplementary material available at 10.1186/s12943-023-01791-1.

## Background

Lung cancer is the leading cause of cancer-related deaths worldwide and remains the most common cancer in China by 2022, according to the latest estimates of global cancer burden data published by the World Health Organization's International Agency for Research on Cancer (IARC) [1]. As the most common type of lung cancer, lung adenocarcinoma (LUAD) accounts for approximately 40% of all cases, and it mostly originates from precancerous lesions, such as atypical adenomatous hyperplasia, progressing to adenocarcinoma in situ and microinvasive adenocarcinoma, and eventually to invasive adenocarcinoma [2–4]. However, despite the great progress of multimodal treatment strategies including targeted therapy, immunotherapy, radiotherapy, and non-invasive surgical resection in recent decades, the five-year overall survival (OS) rate of LUAD is approximately 18% [5–7].

In long-term clinical practice, it has been found that many patients with LUAD have distant metastases at the time of initial diagnosis, and distant metastases in LUAD pose the greatest clinical challenge and may present with a different molecular staging and cellular profile than earlier cancers [8]. Currently, the antiangiogenic targeted therapy for LUAD is considered an effective strategy for the treatment of metastatic LUAD [9], and the monoclonal antibody bevacizumab has been shown to improve response rates and OS in combination with the standard first-line chemotherapy (such as paclitaxel, cisplatin, and carboplatin) for LUAD in phase II and III trials [10]. Other antiangiogenic approaches, such as vascular endothelial growth factor inducers and anticoagulants, are also being investigated [11–13]. Studies have shown that the formation of new blood vessels in the tumor microenvironment is a critical step in the growth of solid tumors and a prerequisite for tumor invasion and metastasis [14, 15]. One of the characteristics of the tumor angiogenesis process is that it stimulates pro-angiogenic factors in endothelial cells [16], which are activated and form various subpopulations. Therefore, a comprehensive and in-depth exploration of the cellular dynamics and molecular features associated with angiogenesis in the ecological niche of metastatic LUAD is required to effectively target key driver genes and develop personalized therapeutic strategies [3, 17, 18].

Insulin-like growth factor 2 (IGF2) mRNA-binding protein 2 (IGF2BP2) is an RNA-binding protein (RBP) with important post-transcriptional regulatory effects on mRNA localization, stability, and translational control [19, 20]. High expression of IGF2BP2 has been shown to be associated with poor prognosis in LUAD, and inhibition of IGF2BP2 expression mitigates LUAD growth, and angiogenesis [21, 22]. Here, this study focuses on exploring the IGF2BP2-mediated mechanism by which angiogenesis of LUAD promotes its metastasis based on single cell analysis. The results of the study may provide new targets for the treatment of LUAD.

## Methods and materials

### Data source

The raw expression matrix for single-cell RNA sequencing (scRNA-seq) of 11 cases of distal normal lung tissues, 11 cases of primary LUAD tissues, and 4 cases of metastatic LUAD tissues was acquired from the Gene Expression Omnibus (GEO) database (http://www.ncbi.nlm.nih.gov/geo/), under the accession number GSE131907 [3]. Sequencing data from metastatic brain tissue samples, normal/metastatic lymph node tissue samples, and pleural effusion samples were excluded from this study.

Furthermore, this study collected 2 cases of LUAD tissue samples with high CD34 expression and 2 cases with low CD34 expression. This study was approved by the Ethics Committee of Yantai Yuhuangding Hospital of Qingdao University (2022–383) and written informed consent was obtained from all patients or their legal guardians who participated in the study.

High-throughput gene expression data from LUAD tissues and normal lung tissues were extracted from The Cancer Genome Atlas (TCGA) data portal (https://tcga-data.nci.nih.gov/tcga). The RNA-seq data from the Illumina HiSeq RNASeq platform (HTSeq-count) consisted of 502 LUAD samples and 49 adjacent non-cancerous samples. Clinical information of LUAD patients was collected from the TCGA database. From the GSE72094 dataset, expression profiling by array and survival information of 442 LUAD patients were acquired [23].

### Tissue immunofluorescence

LUAD tissues with low or high CD34 expression were embedded and sectioned (6 μm thickness), then routinely dewaxed in xylene and alcohol, antigen repaired with citric acid and then washed three times with distilled water for 3 min each time. The sections were incubated with 1% bovine serum albumin (BSA) for 30 min at room temperature. The primary antibody against CD34, EPCAM, IGF2BP2, or FLT4 (Proteintech, Wuhan, China) was diluted with 1% BSA and incubated overnight at 4 ℃. The next day, the sections were washed three times with phosphate buffered saline (PBS) for 3 min each time. Then, the sections were diluted with 1% BSA as fluorescent secondary antibody (Proteintech) and incubated for 1 h at 37 ℃ in a temperature chamber away from light. The sections were washed 3 times with PBS under closed conditions for 3 min each time, and were blocked by adding glycerol diluted Hoechst 33342 (10 μg/mL) (Sigma-Aldrich, St. Louis, Missouri, USA) and photographed by a laser confocal microscope (Nikon, Tokyo, Japan). Fluorescently labeled primary antibodies diluted with 1% BSA were added between washing away the secondary antibodies and adding nucleation reagents, and then the sections were washed three times with PBS for 3 min each time. In the case of non-fluorescently labeled secondary antibodies with L-Lactyllysine, 488-labeled sheep anti-rabbit secondary antibodies were added after incubating the primary antibodies for another 30 min.

### IG2BP2 mRNA in situ hybridization and immunofluorescence co-staining

RNA in situ hybridization was performed according to the kit instructions (Beyotime, Shanghai, China). Firstly, paraffin sections were routinely dewaxed, and proteinase K was briefly digested for 5 min in a 37 ℃ incubator, closed and denatured, then the probes (Sigma-Aldrich) were diluted and mixed with biotin according to the kit instructions, and then added dropwise to the sections for hybridization. Next, the sections were washed for 3 × 15 min, and finally the nuclei were stained with hoechst 33342. For co-staining with other proteins, after probe hybridization, the corresponding fluorescently labeled secondary antibodies were added, and since the corresponding protein would be reduced after proteinase K digestion, the antibody concentration was increased and incubated for 30 min at 37 ℃ in the incubator, followed by nucleation.

### Cell clustering and differentially expressed genes (DEGs)

Firstly, data preprocessing and quality control of scRNA-seq were conducted. Cells without any gene expression, or with > 10% mitochondrial gene expression and < 10% ribosomal gene expression were removed. The Seurat package [24] was then used for cell clustering of scRNA-seq data and to visualize clusters using the uniform manifold approximation and projection (UMAP) package [25]. Cell clusters characterized by similar marker genes were combined into one cell type and subsequently marker genes for known cell types were used to define the partitioned cell clusters. In addition, DEGs in each cell type of control, primary LUAD, and metastatic LUAD tissues were identified by the "FindAllMarkers" function of the Seurat package. Differences associated with adjusted *P* < 0.05 were considered significant.

### Gene regulatory network analysis

The Single Cell Regulatory Network Inference and Clustering (SCENIC) algorithm was developed to evaluate regulatory network analysis associated with transcription factors (TFs) and to discover regulators (*i.e.,* TFs and their target genes) in individual cells. To reconstruct gene regulatory networks for control lung, primary, and metastatic LUAD from scRNA-seq data, we performed SCENIC analysis, which utilizes co-expression modules between TFs and candidate target genes, as well as a database of DNA binding patterns of TFs to infer important gene regulation of TFs [26, 27]. Regulon modules based on regulon crosstalk (correlation between regulons and regulators) were determined by the connection specificity index (CSI), which ranks the importance of regulons and mitigates the effect of non-specific interactions, and were visualized based on the ComplexHeatmap package [28].

### Pseudotime trajectory analysis

The Monocle 3 package (https://cole-trapnell-lab.github.io/monocle3) was applied to sort cells along the pseudotime trajectory [29]. After clustering the cells using the above mentioned method, the dimensionality was reduced and the results were visualized using the UMAP method. Subsequently, cells were sorted according to their progression through the developmental program. In this study, single-cell trajectory analysis of cell subtypes was performed as needed.

### Functional enrichment analysis

Gene Ontology (GO) and Kyoto Encyclopedia of Genes and Genomes (KEGG) pathway analysis was used to determine the biological significance of each cell type. GO and KEGG pathway analyses were performed using the clusterProfiler package based on marker genes for each cell subpopulation [30]. In addition, Gene Set Enrichment Analysis (GSEA) [31] was performed on the DEGs using “c2.cp.kegg.v7.0.symbols.gmt” from the MSigDB database [32] as a background set. GSEA was performed using the clusterProfiler package and *P* < 0.05 was considered significant.

### Cell culture and transfection

Human LUAD cell lines A549 and NCI-H1299 (American Type Culture Collection, Manassas, Virginia, USA) were maintained in Dulbecco’s modified Eagle medium (GIBCO, Gaithersburg, MD, USA) with 10% fetal bovine serum (FBS; GIBCO) and 5% penicillin streptomycin double antibody (GIBCO). Human umbilical vein endothelial cells (HUVECs) (ATCC) were cultivated in EGM™-2 endothelial cell growth medium (Lonza, Basel, Switzerland). All the cells were grown in a 5% CO_2_ incubator at 37 °C.

Small interfering RNA (siRNA) of IGF2BP2 (si-IGF2BP2; sequence: 5’-GGGACCAAGAUAACAAUCUTT-3’ (sense); 5’-AGAUUGUUAUCUUGGUCCCTT-3’ (antisense)) and negative control (si-NC) (GenePharma, Shanghai, China) were synthesized and transfected into LUAD cells through Lipofectamine RNAiMAX transfection reagent (ThermoFisher Scientific, Waltham, Massachusetts, USA).

### Reverse transcription quantitative PCR (RT-qPCR)

Total RNA was extracted via TRZOL reagent (Invitrogen, Carlsbad, California, USA), and complementary DNA was synthesized by a reverse transcription kit (Invitrogen). RT-qPCR was implemented using SYBR Green Master Mix (Solarbio, Beijing, China) and ABI QuantStudio 6 Flex Real-Time PCR System (Applied Biosystems, Carlsbad, USA). Primer sequences were as follows: IGF2BP2, 5’-AGTGGAATTGCATGGGAAAATCA-3’ (forward), 5’-CAACGGCGGTTTCTGTGTC-3’ (revere); GAPDH, 5’-GGAGCGAGATCCCTCCAAAAT-3’ (forward), 5’-GGCTGTTGTCATACTTCTCATGG-3’ (revere). Relative expression was calculated with 2^−ΔΔCt^ method.

### Wound healing assay

LUAD cells were seeded onto 6-well culture plates. Following monolayer formation, a 10 μL pipette tip was used to make a straight scratch. The cells were gently washed by PBS and subsequently replenished by serum-free medium. Images were photographed at 0 h, 24 h, and 48 h under a microscope (Olympus, Tokyo, Japan).

### Transwell assay

Cell invasion assay was conducted utilizing Transwell chambers (Corning Costar, Tewksbury, MA, USA). The upper chamber membrane was coated with Matrigel (BD Bioscience, San Diego, CA, USA). 3 × 10^4^ LUAD cells were plated to the upper chamber of each insert with 300 μL serum-free medium. In addition, the bottom chamber was added with 500 μL medium containing 10% FBS. After 48-h incubation, invasive cells in the bottom chamber were fixed by 10% methanol and stained by 0.1% crystal violet. Invasive cells were photographed under a microscope (Olympus).

### Tube formation assay

Culture medium from LUAD cells was harvested as conditioned medium and utilized for HUVEC culture. HUVECs were plated to Matrigel-coated 96-well plates (1 × 10^4^ cells/well) and cultivated with serum-free medium for 6 h. Next, the HUVECs were maintained with conditioned medium for 6 h. Branch points and capillary length were calculated by use of ImageJ software.

### Analysis of intercellular communication

To further explore the interaction relationships between different cell subpopulations, a cellular communication analysis was performed on endothelial cell subpopulations and LUAD cell subpopulations using the iTALK package [33]. This package matched and paired these genes through the ligand-receptor database by identifying genes that were differentially expressed in cell clusters to find important intercellular communication events. Receptor-ligand interactions were determined using the protein–protein interactions from the STRING database [34].

### Molecular docking

Possible protein-mRNA binding sequences were predicted using the catRAPID omics v2.0 [35]. Molecular docking was performed using the Hex 8.0.0 [36] to test the feasibility of ligand-receptor interactions. Docking models were visualized using Pymol [37]. Docking energies less than 0 indicate that the two have binding potential, and the lower the energy, the higher the binding potential.

### m.^6^A methylated RNA immunoprecipitation-qPCR (MeRIP-qPCR)

Total RNA was extracted from LUAD cells, followed by treatment with DNase (Sigma-Aldrich) to remove genomic DNA. Next, RNA was purified and fragmentated, and the fragments were incubated with m^6^A primary antibody for immunoprecipitation by use of a Magna MeRIP™ m^6^A kit (Millipore, Boston, Massachusetts, USA). Enriched m^6^A modified mRNA was measured via RT-qPCR.

### Western blot

Cells were lysed in RIPA buffer (Beyotime), and protein was fractionated via sodium dodecyl sulfate–polyacrylamide gel electrophoresis, followed by transference onto polyvinylidene fluoride membrane (Millipore). The membrane was blocked in 5% nonfat milk, and subsequently probed with primary antibody against IGF2BP2 (1/2000; ab124930; Abcam, Massachusetts, USA), FLT4 (1/1000; ab243232; Abcam), PI3K (1/1000; ab302958; Abcam), AKT (1/10000; ab179463; Abcam) or β-actin (1/1000; ab8227; Abcam) overnight at 4 °C. Next, the membrane was incubated in horseradish peroxidase-labeled secondary antibody for 2 h at room temperature. Protein band was developed through enhanced chemiluminescence (Millipore), and grey value was quantified with ImageJ software.

### Data analysis and statistics

In this study, all the analyses were performed based on the Bioinforcloud platform (http://www.bioinforcloud.org.cn) and GraphPad Prism software (version 9.0.1; San Diego, CA, USA). Significant difference between two groups was evaluated with Student’s t-test, with one- or two-way ANOVA followed by the Bonferroni post hoc test for comparing between ≥ 3 groups. Correlation analysis was carried out using Pearson or Spearman correlation test. The "Survival" package was performed for survival analysis. Time-independent receiver operating characteristic (ROC) curves were conducted via the "timeROC" package. A nomogram model was built by use of the "rms" package, and was evaluated through calibration curves. *P* < 0.05 was set as statistically significant.

## Results

### A global single-cell landscape of primary and metastatic LUAD

**Fig. 1:**
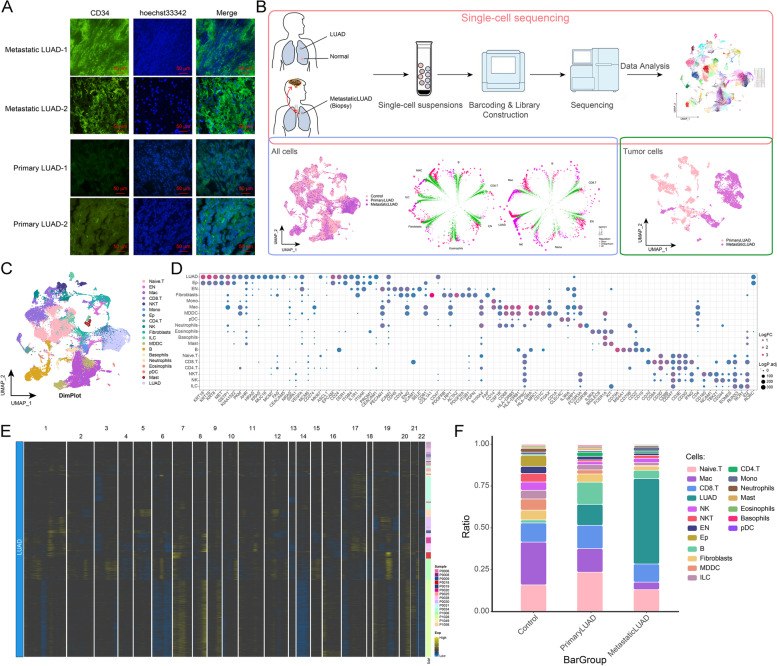
Construction of a global single-cell landscape of primary and metastatic LUAD. **A** Multiplex immunofluorescence map demonstrating CD34 expression in primary and metastatic LUAD tissues. Bar, 50 μm. **B** Flow chart of this study. The global single-cell landscape of primary and metastatic LUAD was constructed based on single-cell technology. **C** The single-cell atlas mapping cell types. **D** Bubble map showing cell marker genes for cell annotations. **E** Heat map showing copy number variation results to further validate the accuracy of cell annotation in the presence of multi-copy or low-copy number events in LUAD cells. **F** The difference in cell abundance between control, primary and metastatic LUAD

In this study, CD34 expression was used to characterize the pathological status of tumor angiogenesis [38]. Immunofluorescence results showed that CD34 expression was higher in metastatic LUAD than in primary LUAD (Fig. 1A). The analytical flow of this study is illustrated in Fig. 1B. We obtained scRNA-seq data of 11 distal normal lung tissues, 11 primary LUAD tissues, and 4 metastatic LUAD tissues from the GSE131907 dataset to further explore the ecological atlas both in primary and metastatic LUAD. After data pre-processing and quality control (Supplementary Fig. 1A-C), a total of 93,610 high-quality single-cells were captured and clustered into 19 cell types, including LUAD cells (LUAD), endothelial cells (En), macrophages (Mac), and others (Fig. 1C). The markers positively expressed in cell clusters were consistent with the gene signatures published by recent scRNA-seq and laboratory research, amongst others [3], and were consistent with the phenotypic characteristics of the corresponding cells (Fig. 1D). Multiple or low copy number events (*i.e.*, malignant copy number variation events) occurred in LUAD tumor cells (Fig. 1E). Further analysis revealed that metastatic LUAD represented a multicellular ecosystem distinct from those of control lung and primary LUAD (Fig. 1F). Notably, a dramatic increase in LUAD cell abundance was observed in metastatic LUAD. In summary, we initially constructed a global single-cell landscape of primary and metastatic LUAD by single-cell analysis and explored the altered cellular ecology in different sample types.

### Clonal evolutionary trajectory of metastatic LUAD tumor cells

**Fig. 2:**
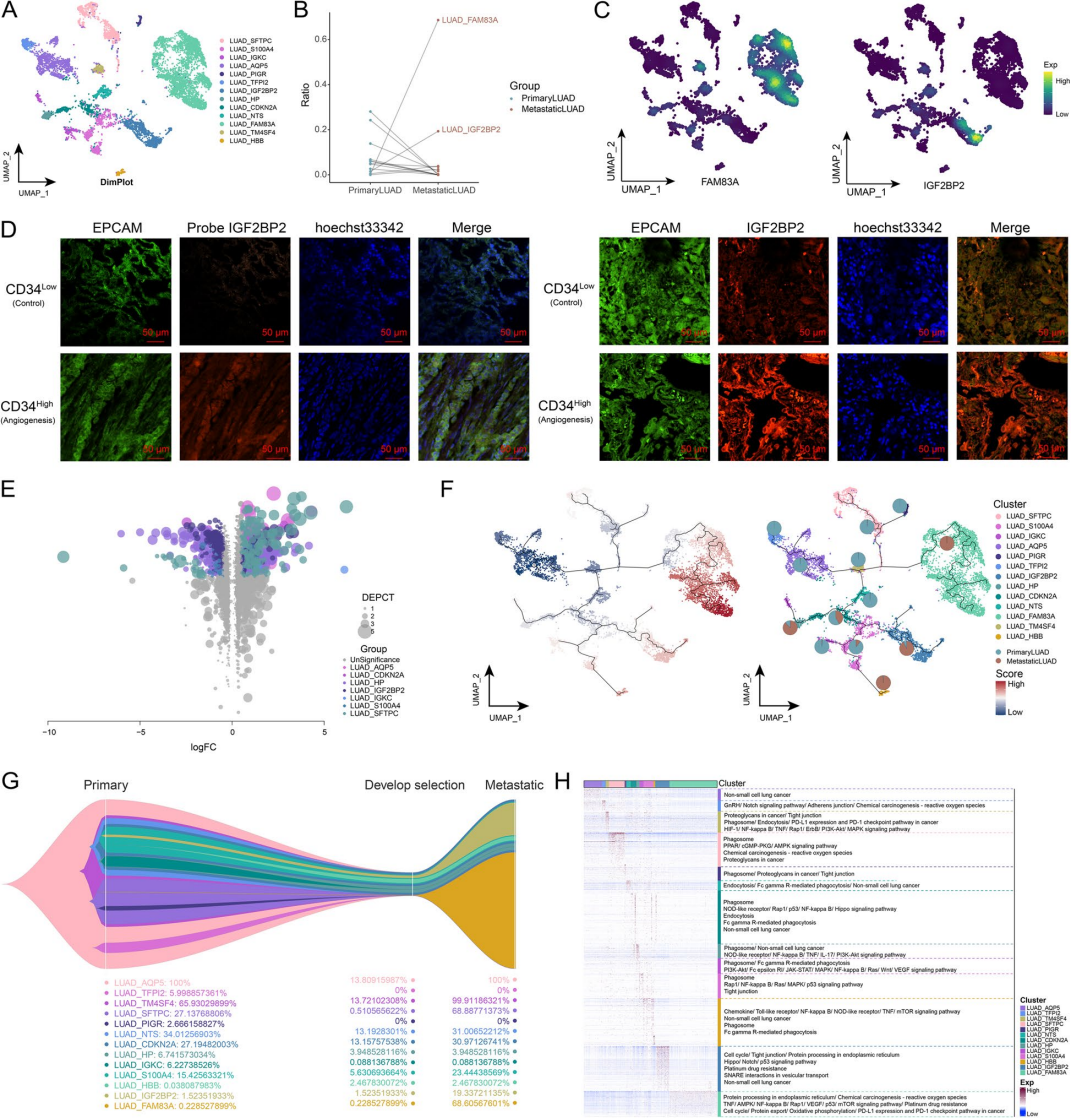
Clonal evolutionary trajectory of metastatic LUAD cells. **A** The single cell atlas showing tumor cell subpopulations. **B** Dotted line graph showing the ratio of distinct tumor cell subpopulations in control, primary and metastatic LUAD. **C** The single-cell atlas mapping the expression of FAM83A and IGF2BP2 in tumor cells. **D** Fluorescence assay-fluorescent probe assay showing IGF2BP2 in LUAD tissues with high or low CD34 expression. Bar, 50 μm. **E** Volcano map showing differentially expressed genes in malignant cells and their subpopulations between metastatic and primary LUAD. **F** The single-cell atlas mapping the pseudotime values and evolutionary trajectory. **G** Evolutionary tree showing the evolutionary trajectory of tumor cells. **H** Heat map-pathway showing signaling pathways involved in temporally relevant genes

The process of clonal evolution of tumor cells is often critical in cancer development [39]. Therefore, to explore the clonal evolutionary trajectory of metastatic LUAD cells, LUAD cell types were first analyzed. Notably, LUAD cells were highly abundant in metastatic LUAD compared to primary tumors, so we performed subpopulation analyses of LUAD cells and constructed a single-cell atlas (Fig. 2A). In exploring the cellular ecology of LUAD cell subpopulations in primary and metastatic LUAD, the abundance of the LUAD_FAM83A and the LUAD_IGF2BP2 subpopulations was found to be significantly higher in metastatic LUAD than in primary tumors (Fig. 2B). The expression of FAM83A and IGF2BP2 was subsequently demonstrated in single-cell profiles (Fig. 2C), and fluorescence results proved that IGF2BP2 expression was significantly higher in LUAD tumors with high CD34 expression than those with low CD34 expression (Fig. 2D), revealing the close relationships between IGF2BP2 and angiogenesis. To further explore the potential molecular mechanisms of metastasis, DEGs in LUAD cell subpopulations between primary and metastatic LUAD were identified (Fig. 2E). According to pseudotime trajectory analysis on LUAD cells (Fig. 2F), an evolutionary trend from primary to metastatic LUAD was identified, which demonstrated the potential structural changes of LUAD cell subpopulations from primary LUAD to natural selection and metastatic disease (Fig. 2G). The signaling pathways and biological functions involved in the proposed time-related DEGs were explored by the functional enrichment analysis, and the LUAD_IGF2BP2 subpopulation was significantly enriched by vesicle synthesis, and secretion of signals (Fig. 2H). In summary, this study inferred the origin and clonal evolution trajectory of metastatic LUAD cell subpopulations and identified the genes involved in the evolutionary process and their biological functions.

### Endothelial cell landscape of metastatic LUAD

**Fig. 3:**
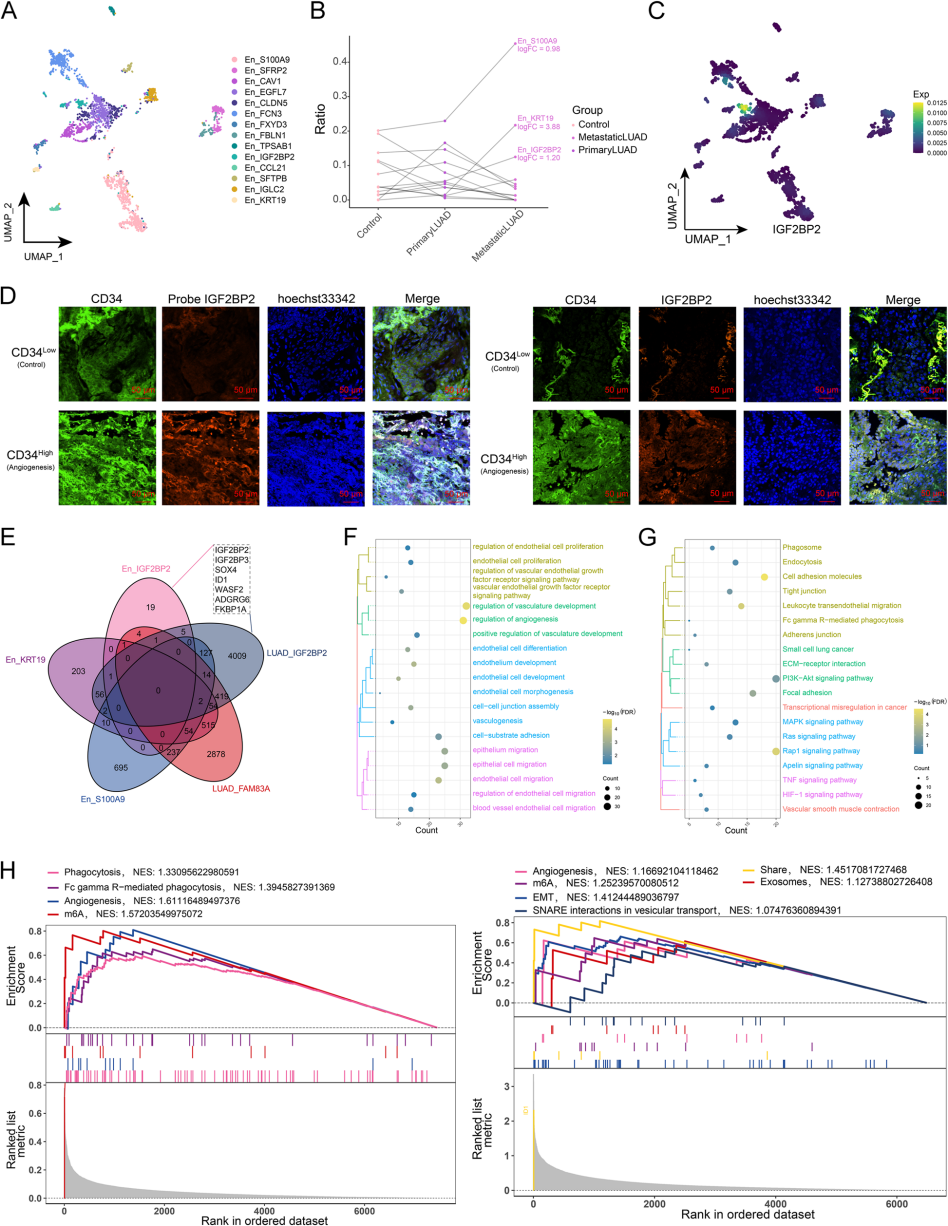
Endothelial cell landscape in metastatic LUAD. **A** The single-cell atlas showing endothelial cell subpopulations. **B** Dotted line graph showing changes in abundance of endothelial cell subpopulations in control, primary and metastatic LUAD. **C** The single-cell atlas mapping the expression of IGF2BP2 in endothelial cells. **D** Immunofluorescence assay-fluorescent probe assay showing IGF2BP2 expression in endothelial cell subpopulations with high and low CD34 expression. Bar, 50 μm. **E** Shared markers of specific cell subpopulations (LUAD_IGF2BP2 and En_IGF2BP2). **F** Cluster-bubble plots demonstrating biological functions shared by specific cell subpopulations. **G** Cluster-bubble plots demonstrating KEGG pathways shared by specific cell subpopulations. **H** GSEA plots demonstrating the significant activation of biological signals related to secretion, secretion and synthesis of vesicles and phagocytosis of contents diffusion in specific cell subpopulations En_IGF2BP2 (left) and LUAD_IGF2BP2 (right)

Cancer progression is characterized by the activation of endothelial cells which in turn promotes tumor angiogenesis. Therefore, endothelial cell subpopulations were identified in the single-cell atlas (Fig. 3A), and the unique cellular ecology of each endothelial cell subpopulation was observed both in primary and metastatic LUAD. Notably, the significant increase in En_S100A9, En_KRT19, and En_IGF2BP2 subpopulations was observed during the progression of primary LUAD to metastasis (Fig. 3B). The simultaneous expression of IGF2BP2 both in the LUAD_IGF2BP2 and the En_IGF2BP2 subpopulations (Fig. 3C, D), indicated the interactions between the two subpopulations. Subsequently, marker genes shared by the LUAD_IGF2BP2 and En_IGF2BP2 subpopulations were further identified, including IGF2BP2, IGF2BP3, SOX4, ID1, WASF2, ADGRG6, and FKBP1A (Fig. 3E). Enrichment analysis (Fig. 3F, G) revealed that these specific cell subsets exhibited the significant activation of critical biological functions such as endocytosis. Further GSEA analysis revealed that angiogenesis, exosome, epithelial mesenchymal transition (EMT), and N^6^-methyladenosine (m^6^A) were notably enriched in the specific cell subpopulations (Fig. 3H). Based on these findings, we tentatively constructed the endothelial cell landscape of metastatic LUAD.

### Targeting IGF2BP2 mitigates migration, invasion, and angiogenesis in LUAD cells

**Fig. 4:**
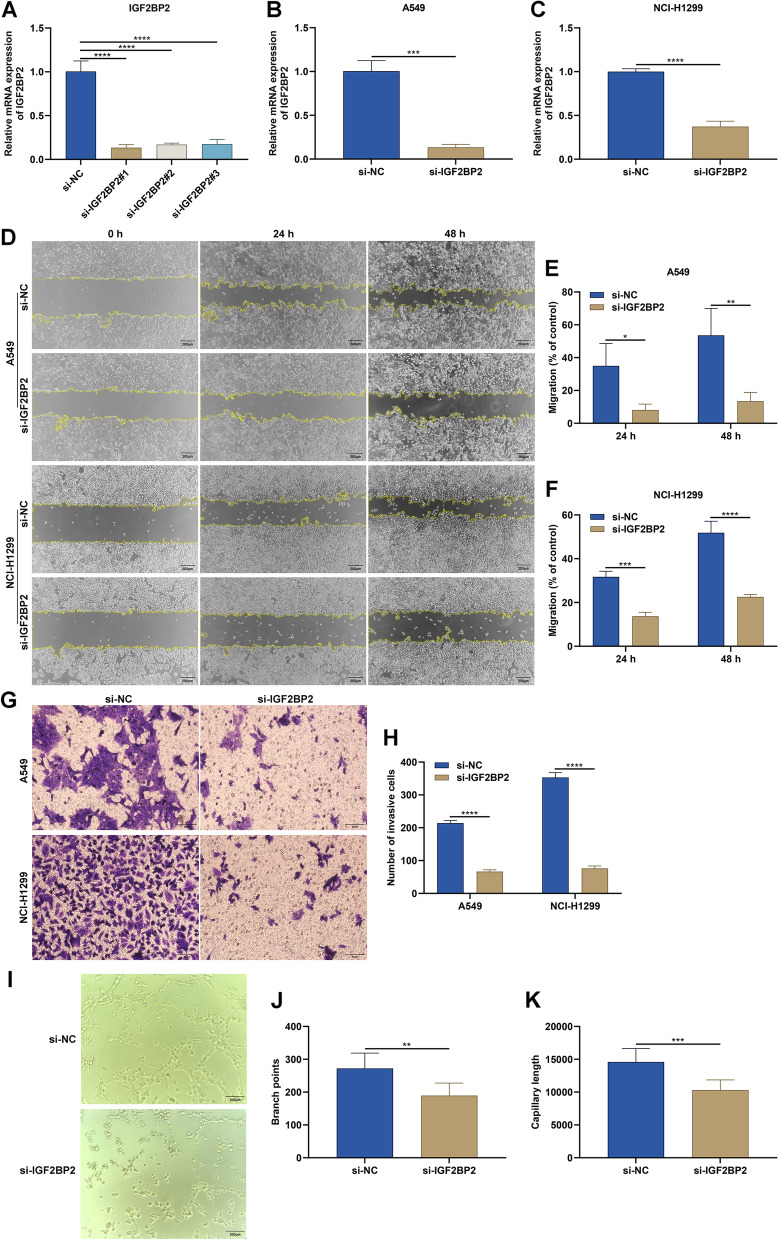
Targeting IGF2BP2 mitigates migration, invasion, and angiogenesis in LUAD cells. **A** Selection of the optimal siRNAs targeting IGF2BP2 through RT-qPCR. **B**, **C** Verification of IGF2BP2 mRNA expression in A549 and NCI-H1299 cells after transfection with si-NC or si-IGF2BP2. **D**-**F** Wound healing scratches at 0 h, 24 h, and 48 h for A549 and NCI-H1299 cells transfected with si-NC or si-IGF2BP2. Bar, 200 μm. **G**, **H** Transwell assay for detecting invasive A549 and NCI-H1299 cells after transfection with si-NC or si-IGF2BP2. Bar, 50 μm. **I**-**K** Images of HUVECs cultured with conditioned medium from A549 cells transfected with si-NC or si-IGF2BP2, and quantification of branch points and capillary length. Bar, 200 μm. *P < 0.05; **P < 0.01; ***P < 0.001; ****P < 0.0001

Next, we investigated the influence of IGF2BP2 on biological behaviors of LUAD cells. For transient transfection, three specific siRNAs targeting IGF2BP2 were designed, and among them, si-IGF2BP2#1 had the optimal interference effect in A549 and NCI-H1299 cells (Fig. 4A-C). According to wound healing assay, migration of A549 and NCI-H1299 cells was significantly decreased by si-IGF2BP2 (Fig. 4D-F). Moreover, our transwell assay results demonstrated that si-IGF2BP2 significantly suppressed invasion of A549 and NCI-H1299 cells (Fig. 4G, H). After HUVECs cultured with conditioned medium from si-IGF2BP2-transfeced A549 cells, both branch points and capillary length were significantly attenuated (Fig. 4I-K), indicating that IGF2BP2 knockdown in LUAD cells inhibited angiogenesis.

### LUAD cell-derived exosomes mediate IGF2BP2 transfer to microenvironmental endothelial cells to activate the PI3K-Akt signaling for angiogenesis

**Fig. 5:**
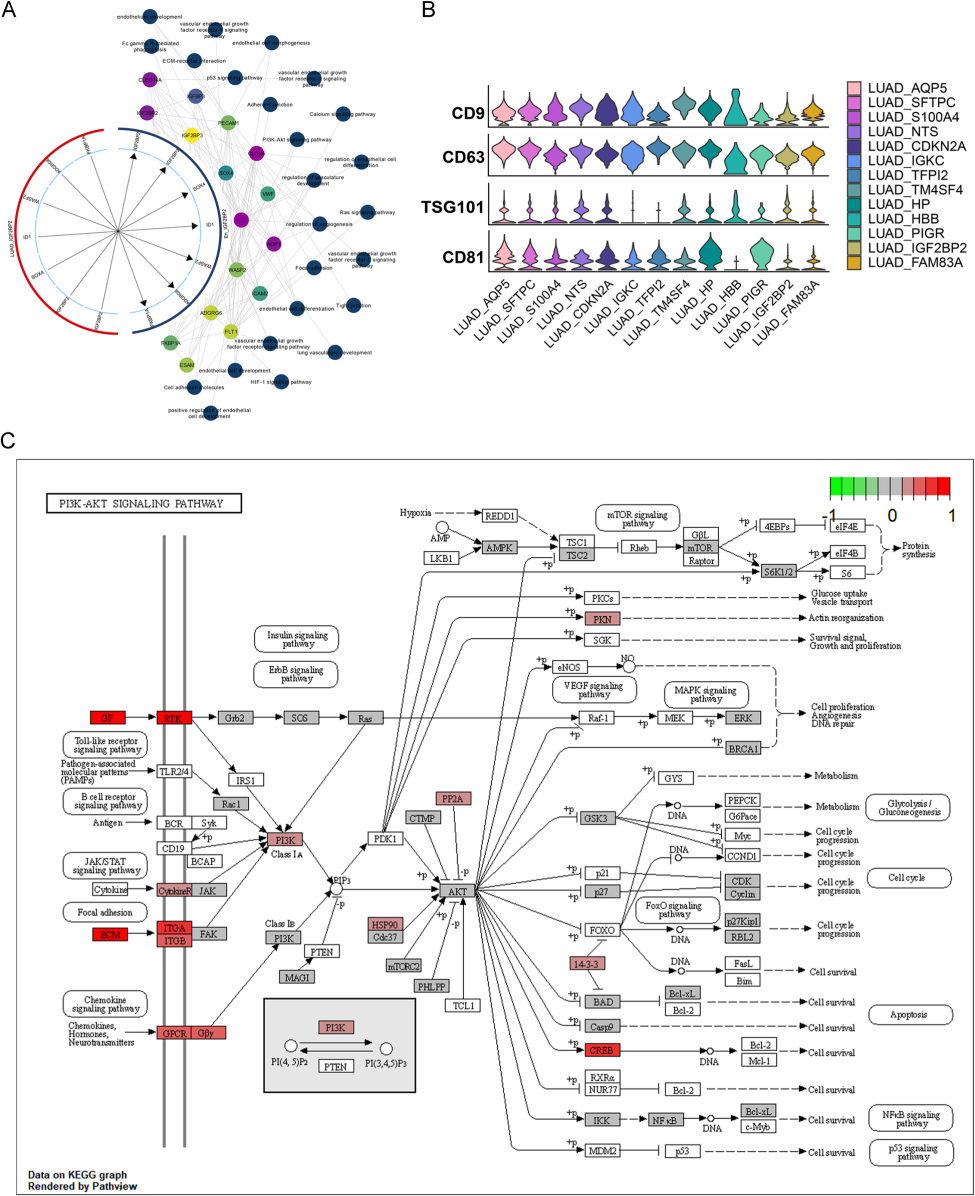
Overexpression of IGF2BP2 in endothelial cells activates the PI3K-Akt signaling to promote angiogenesis. **A** Circle diagram showing the relationships between LUAD_IGF2BP2 ligand genes and En_IGF2BP2 receptor genes. The gene colors characterize their logFC and the pathway colors are aligned with the circles. The first loop genes are shared markers for the LUAD_IGF2BP2 and the En_IGF2BP2 subpopulations, the second loop is a non-shared marker, and the third and fourth loops are signaling pathways. **B** Violin diagram showing the expression pattern of exosomal markers in the LUAD subpopulations. **C** Pathway map showing the PI3K-Akt signaling pathway activated in the En_IGF2BP2 subpopulation

Our above study indicated that metastatic LUAD might achieve angiogenesis through exosomes. Thus, to investigate the mechanism of LUAD cell-derived exosomes transfer to endothelial cells in the tumor microenvironment, we constructed a global regulatory landscape of angiogenesis-specific ecotypes and obtained an integrated regulatory network from the LUAD_IGF2BP2 to the En_IGF2BP2 subpopulations (Fig. 5A). Subsequently, exosome marker genes (CD9, CD63, TSG01 and CD81) were all found to be highly expressed in all LUAD subpopulations, proving the formation of exosomes in LUAD cells (Fig. 5B). Combining the expression of IGF2BP2 both in the LUAD_IGF2BP2 and the En_IGF2BP2 subpopulations, cellular internalization of LUAD cells-derived exosomal IGF2BP2 into endothelial cells was inferred. Furthermore, the PI3K-Akt signaling pathway that confers LUAD angiogenesis and metastasis was found to be activated in the En_IGF2BP2 subpopulation (Fig. 5C). These results indicated that LUAD cells might transmit exosomal IGF2BP2 to endothelial cells to activate the PI3K-Akt signaling and ultimately promote angiogenesis.

### IGF2BP2 mediates the m^6^A modification of FLT4 and activates the PI3K-Akt signaling pathway

**Fig. 6:**
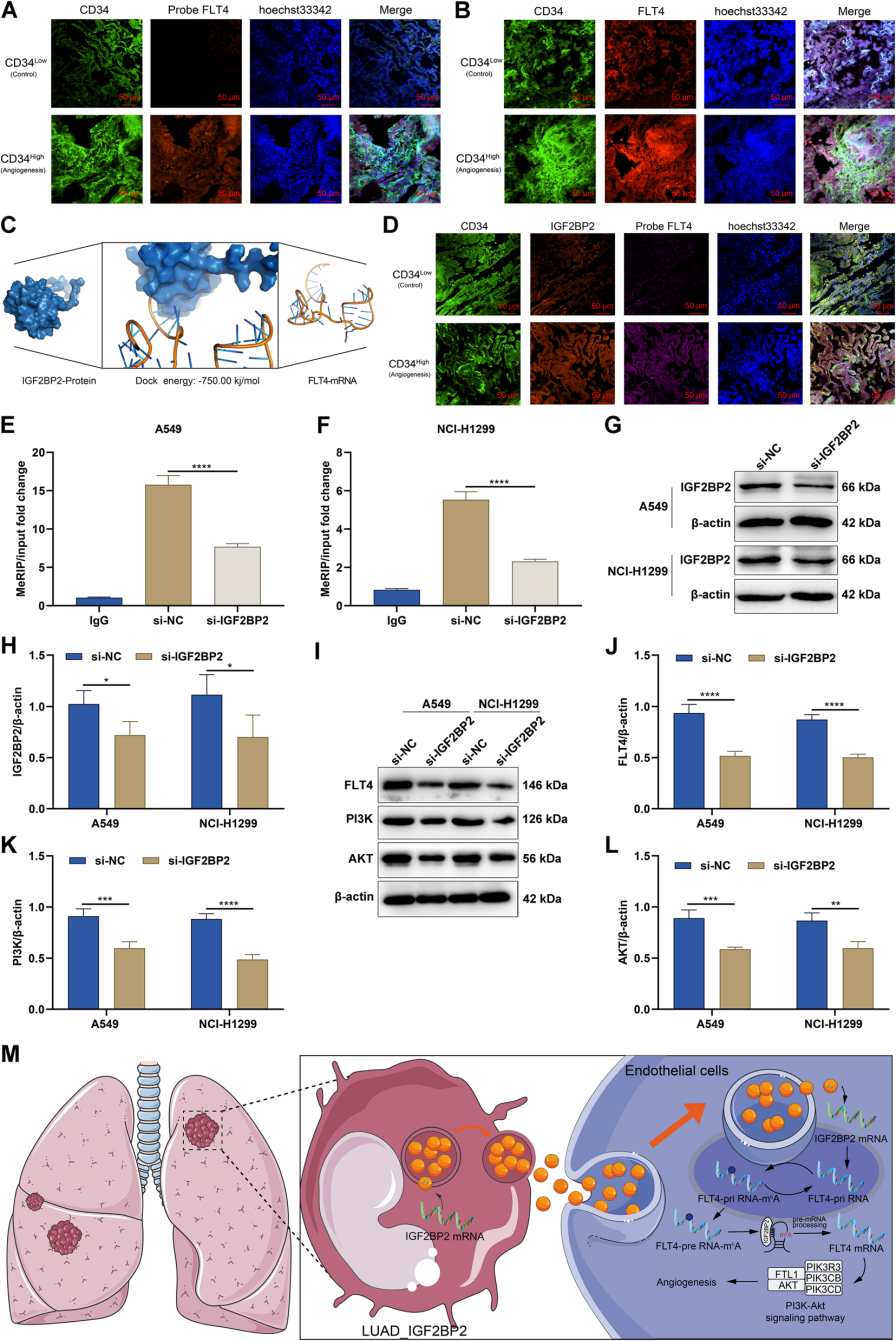
IGF2BP2 activates the PI3K-Akt signaling pathway through mediating the m^6^A modification of FLT4. **A** Fluorescent probe assay for detecting RNA levels of FLT4 in focal endothelial cells of LUAD patients with high or low CD34 expression. Bar, 50 μm. **B** Multiplex immunofluorescence assay for measuring protein levels of FLT4 in focal endothelial cells of LUAD patients with high or low CD34 expression. Bar, 50 μm. **C** Molecular docking for predicting the docking potential of IGF2BP2 protein with mRNA of FLT4. **D** Multiplex immunofluorescence assay for verifying protein levels of IGF2BP2 and RNA levels of FLT4 in focal endothelial cells of LUAD patients with high or low CD34 expression. Bar, 50 μm. **E**, **F** The m.^6^A levels of FLT4 in A549 and NCI-H1299 cells after transfection with si-NC or si-IGF2BP2. **G**, **H** IGF2BP2 protein levels in A549 and NCI-H1299 cells with si-NC or si-IGF2BP2 transfection. **I**-**L** FLT4, PI3K, and AKT expression levels in A549 and NCI-H1299 cells with si-NC or si-IGF2BP2 transfection. **M** The diagram for IGF2BP2-mediated FLT4-PI3K-Akt signaling pathway in regulating angiogenesis during LUAD. *P < 0.05; **P < 0.01; ***P < 0.001; ****P < 0.0001

How IGF2BP2 activates the PI3K-Akt signaling and thus promotes angiogenesis was further probed. FLT4 was identified as a possible gene targeted by IGF2BP2 to activate the PI3K-Akt signaling. Immunofluorescence experiments showed that RNA levels (Fig. 6A) and protein levels (Fig. 6B) of FLT4 were significantly higher in focal endothelial cells of LUAD samples with high CD34 expression than in those with low CD34 expression. The binding potential of IGF2BP2 protein to the mRNA of FLT4 was predicted by molecular docking, and docking energy of -750 kj/mol indicated a great potential for targeted binding between them (Fig. 6C). Multiplex immunofluorescence also demonstrated the co-expression of FLT4 RNA with IGF2BP2 protein (Fig. 6D). Knockdown of IGF2BP2 significantly decreased m^6^A levels of FLT4 both in A549 and NCI-H1299 cells (Fig. 6E, F). In addition, FLT4 protein levels were significantly down-regulated in IGF2BP2-knockout A549 and NCI-H1299 cells (Fig. 6G-J). This demonstrated that IGF2BP2 could up-regulate FLT4 expression through mediating the m^6^A modification of FLT4. Moreover, PI3K and AKT protein levels were significantly decreased in A549 and NCI-H1299 cells with IGF2BP2 knockdown (Fig. 6K, L), indicating that IGF2BP2 was involved in activating the PI3K-Akt signaling pathway. These results suggested that during the clonal evolution of metastatic LUAD, the IGF2BP2-overexpressing LUAD cell subpopulation diffuses IGF2BP2 into the tumor microenvironment and is taken up by endothelial cells by cellular internalization, which subsequently targets to enhance the RNA stability of FLT4 through m^6^A modification, thereby activating the PI3K-Akt signaling pathway, and promoting angiogenesis (Fig. 6M).

### Construction of an IGF2BP2-based prognostic scoring system for LUAD

**Fig. 7:**
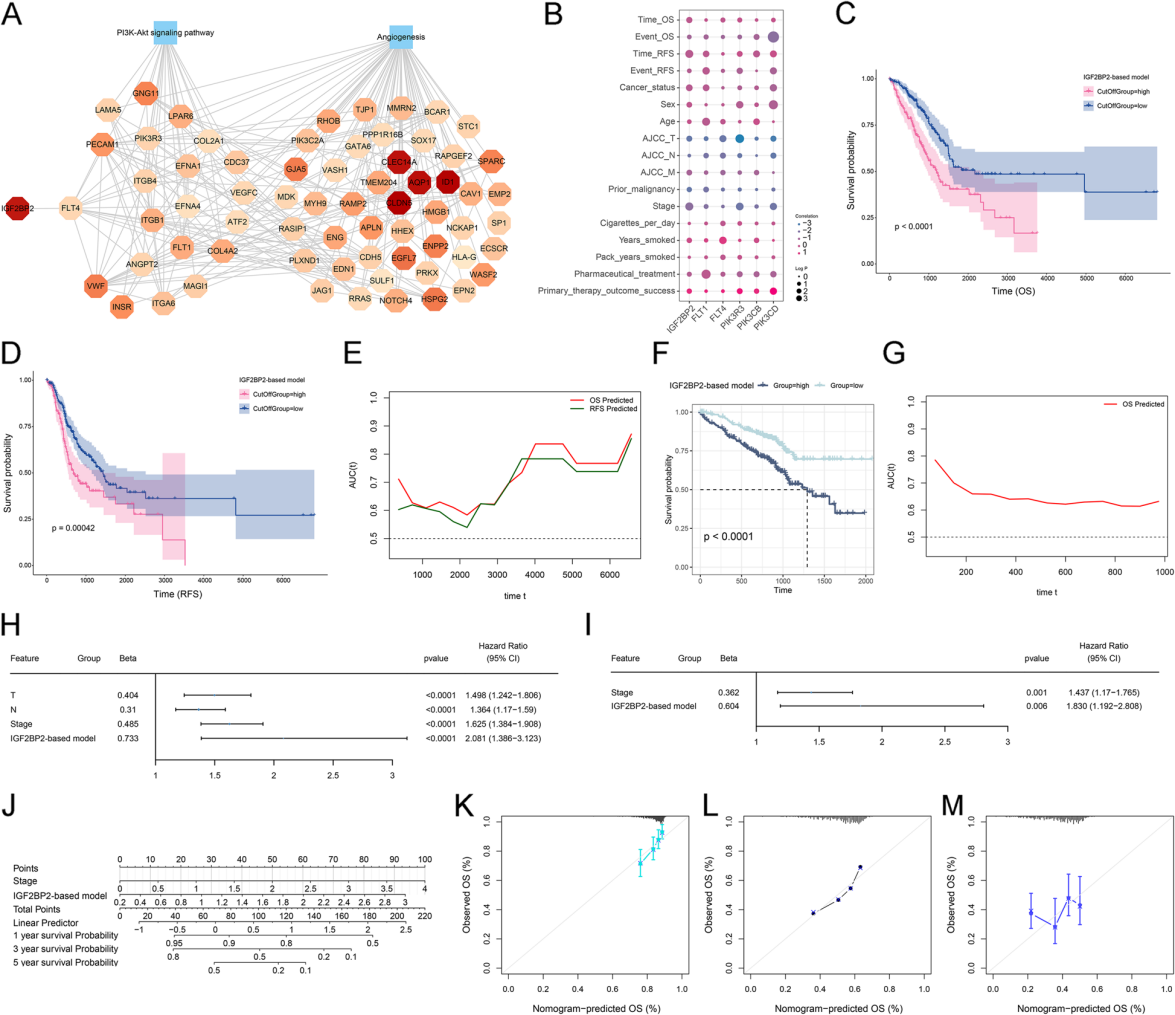
Development of the IGF2BP2-based prognostic scoring system for LUAD. **A** The global regulatory network of IGF2BP2-FLT4-PI3K-Akt signaling-angiogenesis. **B** Bubble plots demonstrating the correlation between the IGF2BP2-FLT4-PI3K-Akt signaling-angiogenesis key genes and clinical indicators in the TCGA-LUAD dataset. **C**, **D** Survival curves of OS and RFS outcomes for the low and high-score patients stratified by the IGF2BP2-based model in the TCGA-LUAD dataset. **E** Time-independent ROC curves for evaluating the performance of the IGF2BP2-based model in predicting OS and RFS outcomes in the TCGA-LUAD dataset. **F**, **G** External validation of OS analysis and time-independent ROC curves in the GSE72094 dataset. **H**, **I** Uni- and multivariate cox regression results on the IGF2BP2-based model and clinical parameters with patient survival in the TCGA-LUAD dataset. **J** Construction of the nomogram composed of the IGF2BP2-based model and stage. **K**-**M** Calibration curves for the nomogram-predicted and actual one-, three- and five-year OS outcomes

We further constructed a global regulatory network of IGF2BP2-FLT4-PI3K-Akt signaling-angiogenesis (Fig. 7A). The potential of these network genes in predicting LUAD prognosis was then evaluated. The association of the key network genes with clinical indicators was explored in the TCGA-LUAD dataset and they were found to be significantly associated with clinical parameters, especially OS and relapse-free survival (RFS) (Fig. 7B). A multivariate cox regression model termed as IGF2BP2-based prognostic scoring system was established based on the key network genes (including IGF2BP2, FLT1, FLT4, PIK3R3, PIK3CB, and PIK3CD), and patients were stratified into low- or high-score groups according to the median value. Survival analyses showed that OS and RFS outcomes were significantly better in the low-score than in the high-score group (Fig. 7C, D). Time-independent ROC curves demonstrated the excellent performance of the model in predicting OS and RFS (Fig. 7E). The IGF2BP2-based prognostic scoring system was proven in an external validation dataset (GSE72094) (Fig. 7F, G). Combining uni- and multivariate-cox regression analyses, it was found that the IGF2BP2-based model and stage were independent risk factors of LUAD prognosis (Fig. 7H, I). We established a nomogram comprising the two independent risk factors (Fig. 7J). Calibration curves demonstrated that the nomogram could accurately predict one-, three- and five-year OS outcomes (Fig. 7K-M). These results indicate the IGF2BP2-FLT4-PI3K-Akt signaling-angiogenesis network plays a crucial role in LUAD prognosis, which provides a theoretical basis for accurate risk stratification of LUAD patients.

### Screening potential small molecule inhibitors of IGF2BP2

Based on the GDSC, and CTRP databases, we predicted potential small molecule inhibitors of IGF2BP2 according to the threshold of correlation coefficient < -0.1 and adjusted *p* < 0.05. Consequently, three small molecule inhibitors shared by the GDSC, and CTRP databases were screened, including trametinib, selumetinib, and dasatinib (Supplementary Fig. 2).

## Discussion

In this study, a global single-cell landscape of primary and metastatic LUAD was constructed and two specific LUAD malignant cell subpopulations (LUAD_IGF2BP2, LUAD_FAM83A) with positive expression of IGF2BP2 and FAM83A, respectively, were captured, and both were highly abundant in metastatic LUAD. In addition, we proposed the mechanism of LUAD cell-derived exosomes mediating IGF2BP2 transfer to microenvironmental endothelial cells to activate the PI3K-Akt signaling for angiogenesis. The genes involved in the mechanisms had prognostic potential and may be potential prognostic markers for LUAD, which will provide a theoretical basis for a more precise assessment of LUAD prognosis.

The family member A gene with sequence similarity 83 (FAM83A) was initially identified as a novel tumor-specific gene, and previous studies demonstrated that it is highly expressed in LUAD and positively associated with poor prognosis, and that it plays an important role in the regulation of LUAD progression [40, 41]. Furthermore, many studies have shown that tumor heterogeneity is responsible for the progression of advanced tumors, and that tumor cells are constantly mutated and differentiated into different subclonal populations during natural selection [14]. The IGF2BP2 and FAM83A expression-positive malignant cell subpopulations identified in this study provide cytological insights into the development of LUAD, where the LUAD_IGF2BP2 subpopulation predominates in metastatic LUAD and is involved in vesicle synthesis, signaling molecule secretion, and exosome-related biological functions, from which we suggested that tumor microenvironment reprogramming during the malignant evolution of metastatic LUAD involves intercellular communication with exosomes as the main means.

We explored subpopulations of endothelial cells and found that subpopulations of endothelial cells positive for S100A9, KRT19, and IGF2BP2 expression (En_S100A9, En_KRT19, and En_IGF2BP2 subpopulations) were highly enriched in metastatic LUAD. Surprisingly, the LUAD_IGF2BP2 subpopulation expressed the same subpopulation marker IGF2BP2 as the En_IGF2BP2 subpopulation, and co-expressed some m^6^A modifiers (IGF2BP3, SOX4, ID1, WASF2, ADGRG6 and FKBP1A); moreover, enrichment analysis revealed that phagocytosis was significantly activated in the En_IGF2BP2 subpopulation. It was inferred that LUAD cell-derived exosomes mediated the delivery of IGF2BP2 to microenvironmental endothelial cells. Further information combined with downstream regulatory levels confirmed that IGF2BP2 acts as an m^6^A methylation reader, enhancing the RNA stability of FLT4, which in turn promotes the PI3K-Akt signaling-endothelial activation cascade. FLT4 encodes the tyrosine kinase receptor for vascular endothelial growth factors C and D, and its expression is significantly upregulated in microvessels of tumors and wounds, however, its role has not been investigated in LUAD [42]. IGF2BP2 is a unique m^6^ A reader that targets a large number of mRNA transcripts and promotes the stability and storage of targeted mRNAs in oncogenic effects [20]. It is well known that m^6^A modification induces oncogenic protein expression, cancer cell proliferation, survival, tumorigenesis and progression [43], and thus the overexpression of m^6^A modification reader (IGF2BP2) is highly likely to promote the progression of LUAD [39]. Although m^6^A modification is one of the common post-transcriptional epigenetic modalities with important roles in tumor development [44], it remains largely uncharted territory [45]. The present study put forward the novel theory that the m^6^A modification regulatory factor (IGF2BP2) is transmitted from malignant cell subclones to microenvironmental endothelial cells via exosomes and is subsequently taken up and forms a distinct endothelial subpopulation, which in turn promotes LUAD angiogenesis and metastasis.

In addition, based on the global regulatory network of IGF2BP2-FLT4 axis regulating the PI3K-Akt signaling pathway to promote angiogenesis, this study constructed an IGF2BP2-based clinical model and found that it was significantly associated with LUAD prognosis. Based on the above findings, it was confirmed that the IGF2BP2-based prognostic scoring model not only helps to assess the prognostic outcomes of patients, but also contributes to an in-depth understanding of the cancer metastasis mechanism, and also provides a profound theoretical basis and scientific foundation for the development of anti-LUAD strategies by targeting IGF2BP2-based m^6^A modification.

## Conclusion

In summary, this study constructs a global single-cell landscape of primary and metastatic LUAD through single-cell transcriptomics analysis and reveals an IGF2BP2-based mechanism in LUAD angiogenesis and metastasis, which provides an opportunity to elucidate the relevant cellular dynamics and molecular features of LUAD metastasis.

## Supplementary Information

**Additional file 1: Supplementary Fig. 1.** Data preprocessing and quality control of scRNA-seq. A, B. Violin plots showing the number of genes detected in each cell (nFeature_RNA), the total number of mRNA molecules detected in the cells (nCount_RNA), and the percentage of mitochondrial gene expression in the total gene expression (percent.mt) (A) before and (B) after quality control. C. Scatter plots showing the distribution of nCount_RNA and percent.mt as well as nCount_RNA and nFeature_RNA to filter low-quality cells.

**Additional file 2: Supplementary Fig. 2.** Prediction of potential small molecule inhibitors of IGF2BP2 based on the GDSC, and CTRP databases.

## Data Availability

The datasets analyzed during the current study are available from the corresponding author on reasonable request.

## Abbreviations

LUAD: Lung adenocarcinoma
OS: Overall survival
IGF2BP2: Insulin-like growth factor 2 (IGF2) mRNA-binding protein 2
scRNA-seq: Single-cell RNA sequencing
TCGA: The Cancer Genome Atlas
DEGs: Differentially expressed genes
UMAP: Uniform manifold approximation and projection
SCENIC: Single Cell Regulatory Network Inference and Clustering
TFs: Transcription factors
GO: Gene Ontology
KEGG: Kyoto Encyclopedia of Genes and Genomes
GSEA: Gene Set Enrichment Analysis
siRNA: Small interfering RNA
RT-qPCR: Reverse transcription quantitative PCR
MeRIP-qPCR: M^6^A methylated RNA immunoprecipitation-qPCR
ROC: Receiver operating characteristic
RFS: Relapse-free survival
