# Supplementary figures and images for: m6A methylation reader IGF2BP2 activates endothelial cells to promote angiogenesis and metastasis of lung adenocarcinoma
Source: Mol Cancer. 2023 Jun 23;22:99. doi: 10.1186/s12943-023-01791-1 (PMC10288689; doi:10.1186/s12943-023-01791-1)

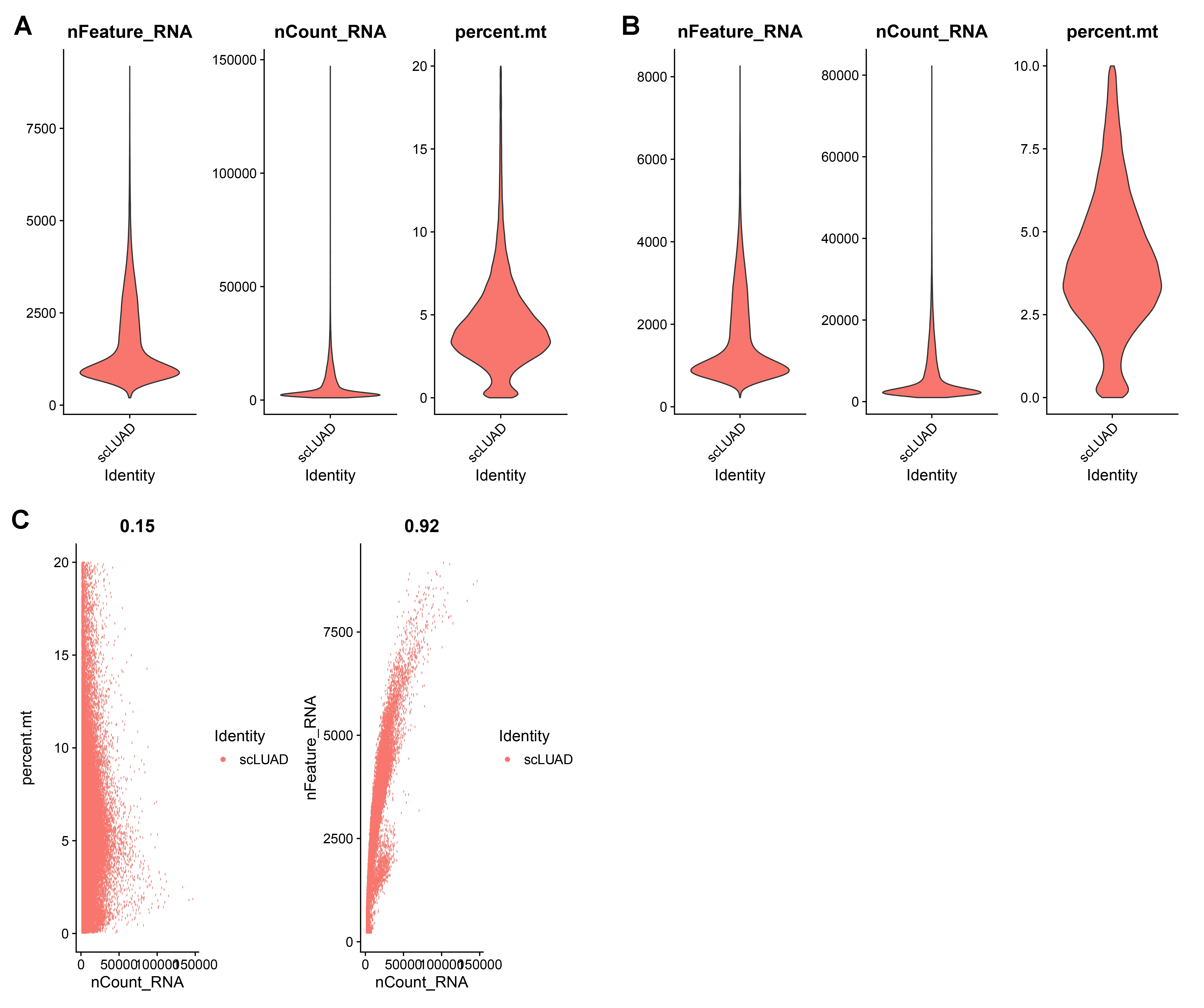

Supplement: Supplementary file 1 — Additional file 1: Supplementary Fig. 1. Data preprocessing and quality control of scRNA-seq. A, B. Violin plots showing the number of genes detected in each cell (nFeature_RNA), the total number of mRNA molecules detected in the cells (nCount_RNA), and the percentage of mitochondrial gene expression in the total gene expression (percent.mt) (A) before and (B) after quality control. C. Scatter plots showing the distribution of nCount_RNA and percent.mt as well as nCount_RNA and nFeature_RNA to filter low-quality cells. [file 12943_2023_1791_MOESM1_ESM.tif]

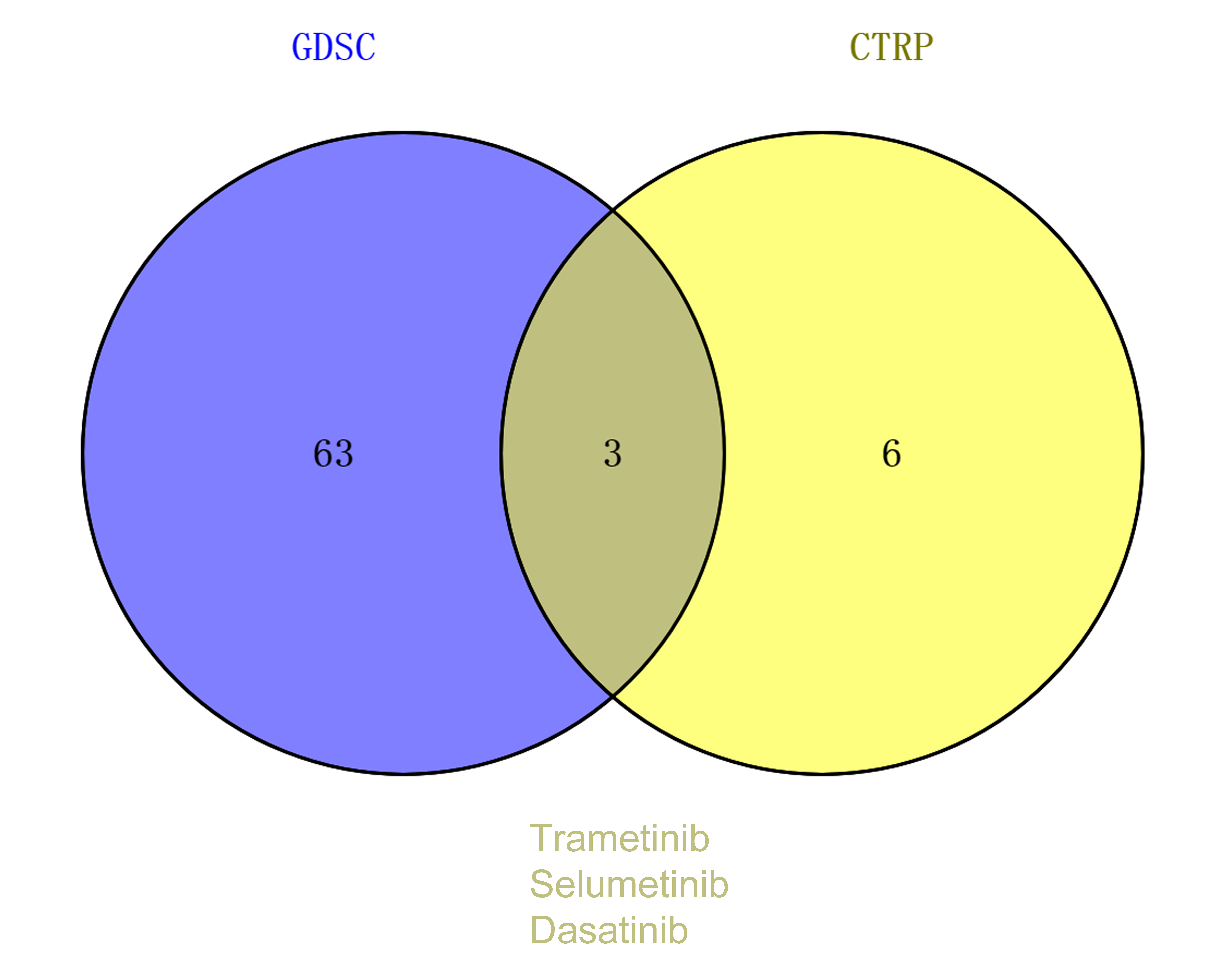

Supplement: Supplementary file 2 — Additional file 2: Supplementary Fig. 2. Prediction of potential small molecule inhibitors of IGF2BP2 based on the GDSC, and CTRP databases. [file 12943_2023_1791_MOESM2_ESM.tif]
